# Immunization With Fc-Based Recombinant Epstein–Barr Virus gp350 Elicits Potent Neutralizing Humoral Immune Response in a BALB/c Mice Model

**DOI:** 10.3389/fimmu.2018.00932

**Published:** 2018-05-01

**Authors:** Bingchun Zhao, Xiao Zhang, Claude Krummenacher, Shuo Song, Ling Gao, Haojiong Zhang, Miao Xu, Lin Feng, Qisheng Feng, Musheng Zeng, Yuting Xu, Yixin Zeng

**Affiliations:** ^1^Guangdong Key Laboratory of Nasopharyngeal Carcinoma Diagnosis and Therapy, State Key Laboratory of Oncology in South China, Collaborative Innovation Center for Cancer Medicine, Department of Experimental Research, Sun Yat-Sen University Cancer Center, Guangzhou, China; ^2^Department of Biological Sciences, Rowan University, Glassboro, NJ, United States; ^3^Department of Molecular and Cellular Biosciences, Rowan University, Glassboro, NJ, United States; ^4^State Key Laboratory of Molecular Vaccinology and Molecular Diagnostics, School of Life Sciences, National Institute of Diagnostics and Vaccine Development in Infectious Diseases, Xiamen University, Xiamen, China; ^5^State Key Laboratory of Molecular Vaccinology and Molecular Diagnostics, School of Public Health, National Institute of Diagnostics and Vaccine Development in Infectious Diseases, Xiamen University, Xiamen, China; ^6^Guiyang City National High School, Guiyang, China

**Keywords:** Epstein–Barr virus, envelope protein, gp350, Fc-based vaccine, neutralizing antibody, intranasal immunization

## Abstract

Epstein–Barr virus (EBV) was the first human virus proved to be closely associated with tumor development, such as lymphoma, nasopharyngeal carcinoma, and EBV-associated gastric carcinoma. Despite many efforts to develop prophylactic vaccines against EBV infection and diseases, no candidates have succeeded in effectively blocking EBV infection in clinical trials. Previous investigations showed that EBV gp350 plays a pivotal role in the infection of B-lymphocytes. Nevertheless, using monomeric gp350 proteins as antigens has not been effective in preventing infection. Multimeric forms of the antigen are more potently immunogenic than monomers; however, the multimerization elements used in previous constructs are not approved for human clinical trials. To prepare a much-needed EBV prophylactic vaccine that is potent, safe, and applicable, we constructed an Fc-based form of gp350 to serve as a dimeric antigen. Here, we show that the Fc-based gp350 antigen exhibits dramatically enhanced immunogenicity compared with wild-type gp350 protein. The complete or partial gp350 ectodomain was fused with the mouse IgG2a Fc domain. Fusion with the Fc domain did not impair gp350 folding, binding to a conformation-dependent neutralizing antibody (nAb) and binding to its receptor by enzyme-linked immunosorbent assay and surface plasmon resonance. Specific antibody titers against gp350 were notably enhanced by immunization with gp350-Fc dimers compared with gp350 monomers. Furthermore, immunization with gp350-Fc fusion proteins elicited potent nAbs against EBV. Our data strongly suggest that an EBV gp350 vaccine based on Fc fusion proteins may be an efficient candidate to prevent EBV infection in clinical applications.

## Introduction

Epstein–Barr virus (EBV), known as human herpesvirus 4, is a highly prevalent virus from the Herpesviridae γ subfamily (1). EBV is the first virus reported to cause tumorigenesis in infected humans (1–3). It is widely spread among human populations, and over 95% of adults carry latent EBV worldwide (4, 5). EBV establishes lifelong latency in infected individuals. Three types of latencies have been described based on the subset of genes expressed in latently infected cells (6). The latent status of EBV is closely linked to the development and progression of many diseases, such as infectious mononucleosis (IM), nasopharyngeal carcinoma (NPC), posttransplant lymphoproliferative disease, EBV-related gastric carcinoma, and Hodgkin lymphoma (7–14). In some southern provinces of China, where NPC is frequent, it has the highest fatality rate among malignancies. Thus, EBV-associated diseases are an important public health concern. In addition, latent EBV infection was recently shown to accelerate the decay of childhood measles and rubella vaccine responses (15). However, at present no EBV vaccine is available. Development of a successful EBV vaccine is difficult due to (i) the lack of appropriate animal models for evaluation of infection, (ii) the poor protection of previous vaccine candidates, and (iii) inadequate investment in vaccine development (16–18).

To initiate EBV infection, several viral envelope glycoproteins are involved in entry into target cells such as epithelial cells or B-lymphocytes (19, 20). Out of these glycoproteins, gp350 is generally considered to be involved in the infection of B-lymphocytes (21). Two cellular receptors, CR2/CD21 and CD35, bind gp350 during entry. The crystal structure of gp350 ectodomain (residues 1–470) showed that about 14 large glycan chains cover the whole surface of gp350 except for the area, which is responsible for binding to CR2/CD21 (22). Despite being the most abundant EBV glycoprotein on virions (23), this glycosylation pattern suggests that native gp350 remains monomeric since its important glycan coverage may prevent protein-protein interaction on the viral envelope (22). Importantly, a monoclonal antibody (mAb72A1), which was raised against gp350, effectively blocks EBV infection of B cells (24–27). Hence, gp350 has been the primary antigen used in the development of EBV prophylactic vaccines (17). Initially, Epstein et al. reported that using purified gp350 as antigen can protect cotton marmosets from developing lymphoma caused by EBV infection (28). In some cases, the immune sera could block infection although neutralizing antibodies (nAbs) were rarely found. This observation implied that most antibodies against purified gp350 probably clear the virus through ADCC or T cell responses (29–31). Polymerization of gp350 on the surface of ferritin nanoparticles significantly increased antigenicity and protected mice against infection by a recombinant vaccinia virus expressing EBV gp350 (32). Candidate vaccines based on gp350 have been tested in clinical trials. Recombinant vaccinia vectors expressing gp350 elicited nAbs against EBV (33). More recently, a recombinant gp350 antigen induced protection from EBV infection in a Phase I/II clinical trial (34). Despite these results, none of these trials have yet reached the final stage of vaccine development. Interestingly, in a Phase II trial performed with seronegative individuals, IM incidence was notably decreased, but EBV infection could not be blocked (35). Besides, recombinant gp350 did not induce nAbs in a cohort of children awaiting kidney transplants, even though the specific antibody titer increased soon after immunization (36).

In Fc-based fusion antigens, the Fc domain of immunoglobulins is fused with the desired protein, which allows the dimerization of the protein and often potently increases its biological and pharmacological characteristics (37–39). The fusion with the Fc domain can remarkably increase plasma half-life and immunogenicity of recombinant antigens. This appears to be due to the combination of a high affinity for the neonatal Fc receptor (FcRn) and a slower clearance due to higher molecular weight (40–42). Besides, the Fc fusion proteins can be easily purified through protein A/G affinity chromatography. Furthermore, the safety of use of Fc in fusion proteins in humans has been clearly established during the development of Fc-based protein drugs and therapeutic mAbs (37, 43). For all these reasons, we believe that an Fc fusion approach is a valid way to improve EBV g350 antigen immunogenicity and efficacy as a prophylactic vaccine.

In this study, we expressed two forms of recombinant EBV gp350 ectodomain fused with the Fc domain of mouse IgG2a (mIgG2a-Fc) in insect cells. We used the N-terminal three domains of gp350 (gp350-ECD_123_) or the full-length gp350 ectodomain (gp350-ECD_FL_). We found that the fusion with Fc did not affect the neutralizing epitope recognized by mAb72A1 or the CR2/CD21 binding site of gp350. The immunogenicity of gp350-ECD_123_ and gp350-ECD_FL_ with and without Fc fusion was compared in BALB/c mice. Infection neutralization assays and antibody-dependent competition indicated that recombinant gp350 fused with Fc domain elicited more potent nAbs compared with non-fusion monomeric gp350 proteins.

## Materials and Methods

### Construction of Recombinant Bacmids With Insertion of gp350 Ectodomain Fused With mIgG2a-Fc

**Figure 1:**
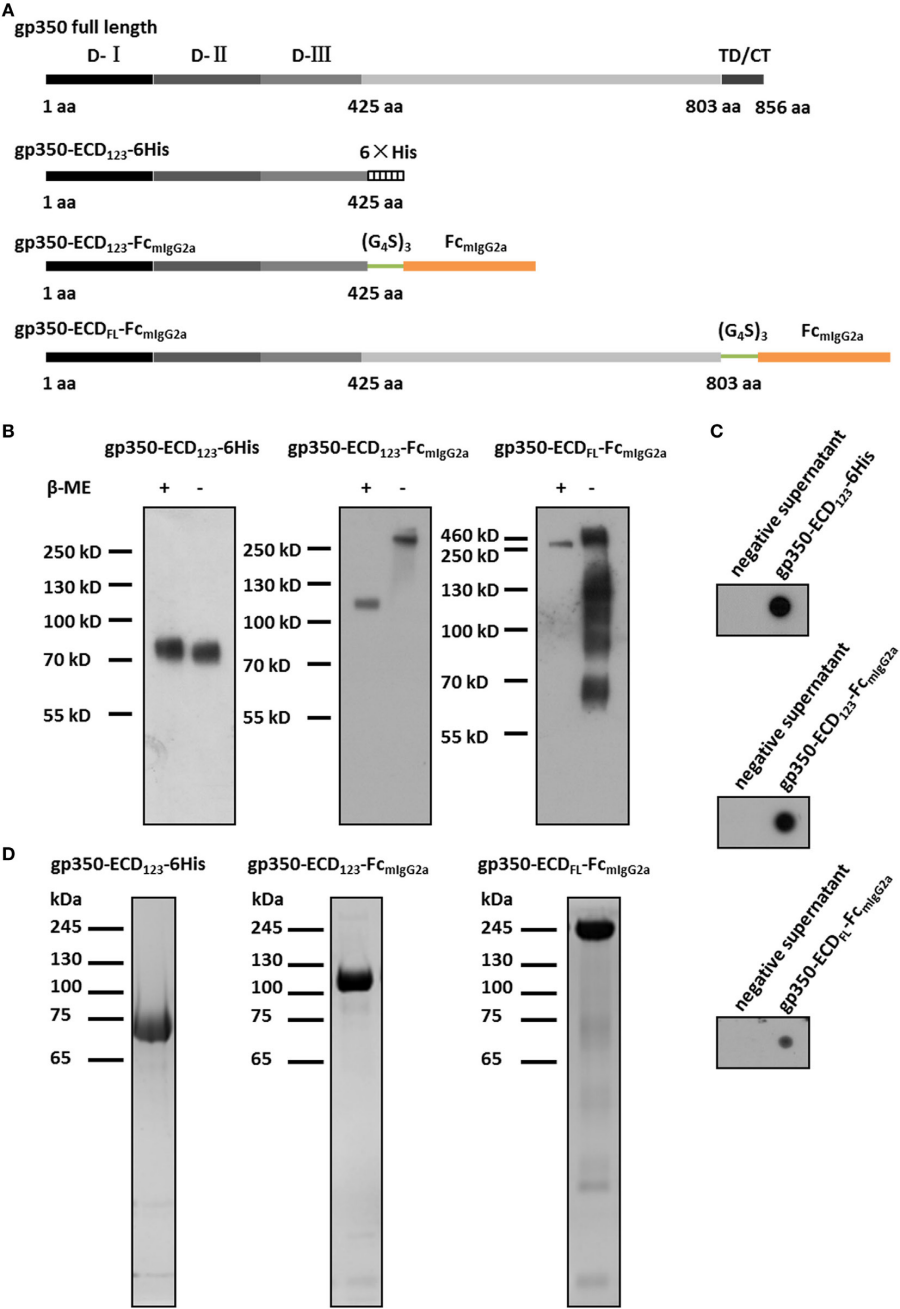
Characterization of Fc-based recombinant gp350 proteins. **(A)** Diagrams for design of Fc-based recombinant gp350 proteins. The extracellular domains (D-I, D-II, and D-III), transmembrane domains, and cytoplasmic tail (TD/CT) are shown in gray. The 6-histidine tag is shown as a hatched box, the mouse IgG2a Fc domain is shown orange, and the glycine–serine (G_4_S)_3_ linker is colored green. **(B)** Western blot of secreted recombinant gp350 constructs under reducing (+β-ME) and non-reducing conditions (−β-ME). gp350-ECD_123_-6His was detected by an anti-His tag antibody, and proteins fused with Fc domain were detected with an anti-mouse Fc antibody. For each fusion protein, the same volume (15 µl) of supernatants from identical passage 1 baculovirus stocks was loaded onto the gels under reducing and non-reducing conditions. Samples were resolved on 10% SDS-PAGE. **(C)** Detection of the indicated recombinant gp350 forms by conformation-dependent anti-gp350 mAb72A1 antibody in the supernatant of bacmid-transfected Sf9 cells using dot blot. Cell culture supernatants from mock-transfected cells were included as negative controls. **(D)** SDS-PAGE analysis of SEC-purified forms of recombinant gp350. Proteins from the peak fractions were resolved on 10% gel under reducing condition and stained with Coomassie brilliant blue.

Two variants of EBV gp350 ectodomain, gp350-ECD_123_ (residues 1–425, corresponding to the first three N-terminal domains) and gp350-ECD_FL_ (residues 1–803, corresponding to the full-length ectodomain) were used. The corresponding sequences from strain M81 were obtained by PCR amplification using specific primers: gp350-ECD_123_-BamH I-F (5′-CGCGGATCCGCCACCATGGTAAGCGCTATTGTTTTATATGTGCTTTTGGCGGCGGCGGCGCATTCTGCCTTTGCGATGGAGGCAGCCTTGCTTGTG-3′) and gp350-ECD_123_-Sal I-R (5′-ACGCGTCGAC GGGTGCCTTGGAGAATATAACCTTGTG-3′) for gp350-ECD_123_, gp350-ECD_FL_-BamH I-F (5′-CGC GGATCCGCCACCATGGTAAGCGCTATTGTTTTATATGTGCTTTTGGCGGCGGCGGCGCATTCTGCCTTTGCGATGGAGGCAGCCTTGCTTGT-3′) and gp350-ECD_FL_-Sal I-R (5′-ACGCGTCGAC GGAGAGGTTTGAGAATCTGG-3′) for gp350-ECD_FL_. These two fragments were inserted into the pFastBac1 vector (Invitrogen) by BamH I/Sal I digestion. To guide the secretion of the Fc fusion proteins and obtain correct glycosylation, the coding sequence of gp64 signal peptide was added at the 5′ end of the gp350 fragment by inclusion in the primers. The coding fragment of mIgG2a-Fc was synthesized as previously reported (44) and added to the 3′ end of the gp350 fragments by Sal I/Xba I digestion. The soluble gp350-ECD_123_ was constructed by adding a C-terminal hexa-histidine tag, and an N-terminal gp64 signal peptide fragment included in the PCR primers. The resulting constructs are shown in Figure 1A.

The recombinant bacmids containing the coding fragments of mIgG2a-Fc fusion protein were constructed according to the manufacturer’s manual. Briefly, DH10B cells were transformed by heat shock with each plasmid constructs. The DH10B cells with positive recombinant bacmids were isolated as white colonies on LB agar plates containing kanamycin, gentamicin, tetracycline, Bluo-gal, and IPTG. Positive colonies were validated by restreaking and the presence of genes encoding fusion proteins was verified by PCR using pUC/M13 Forward and Reverse primers. Finally, the recombinant bacmids were extracted by alkaline lysis, dissolved in TE buffer, and stored at −20°C for subsequent transfection.

### Expression and Purification of Fc Fusion Proteins

The expression of Fc fusion proteins in insect cells followed the bac-to-bac manual of Invitrogen. Briefly, ~8 × 10^5^ Sf9 cells per well were seeded in a 6-well plate and cultured in monolayers in Sf900-III media (Invitrogen) and transfected with recombinant bacmids using Cellfectin reagents (Invitrogen). Expression was validated in passage 1 (P1) baculovirus stocks after 7–10 days by Western blot. The P1 baculoviruses were subjected to two rounds of plaque selection to obtain positive pure recombinant viruses. For protein expression, P1 recombinant viruses were subjected to two cycles of amplification in High 5 cells cultured in suspension in ESF921 medium (Expression system). High 5 cells were seeded at 0.5 × 10^6^ cells/ml and infected with recombinant viruses when the cell density reached 1.5–2.0 × 10^6^ cells/ml. The multiplicity of infection and infection time were optimized for each recombinant fusion protein to maximize protein expression levels. Culture supernatants containing recombinant proteins were harvested 4–5 days posttransfection, and cell debris was removed by centrifugation and 0.45 µm filtration.

All culture supernatants were concentrated 10-fold using Mini Pellicon TFF system (Millipore). Soluble gp350-ECD_123_-6His was purified using HisTrap FF prepacked column (GE Healthcare) with a GE Akta purifier chromatography system. The concentrated supernatants were dialyzed against equilibration buffer (20 mM Tris–HCl, 300 mM NaCl, 10 mM imidazole, pH 7.5), and the HisTrap column was equilibrated with 5–10 column volumes of equilibration buffer. The dialyzed supernatants were applied on the column, which was washed with equilibration buffer until the absorbance reached baseline. Elution was performed using at least five column volumes of elution buffer (20 mM Tris–HCl, 300 mM NaCl, 60 mM imidazole, pH 7.5). For purification of Fc fusion proteins, the concentrated supernatants were loaded on the pre-equilibrated HiTrap Mabselect column (GE Healthcare) after dialyzed against 1× PBS. The bound fusion proteins were eluted using 100 mM sodium citrate, pH 3.0, and neutralized with 1 M Tris–HCl, pH 9.5 (300 µl Tris–HCl buffer per 1 ml elution). All the eluted proteins were subjected to SDS-PAGE and Coomassie staining for purity check.

The purified proteins were immediately dialyzed against 1× PBS and loaded on a Superdex200 increase 10/300 GL prepacked column (GE Healthcare) for further purification. Generally, the samples were concentrated four to five times for loading, and the flow rate was set to 0.5 ml/min. The protein absorbance was monitored at 280 nm. The eluted fractions were checked for purity of fusion proteins using SDS-PAGE, pooled together, and protein concentration was determined using BCA assay kit (Thermo Scientific Pierce) after concentration. Finally, the fusion proteins were aliquoted and stored at −80°C.

### Construction and Purification of Human CR2 (SCR1-2) Fused With MBP Tag

The construction and purification of human CR2 (SCR1-2) fusion protein was performed as described previously (45). Briefly, coding sequence corresponding to residues 1–133 of wild-type human CR2 (SCR1-2) was synthesized and inserted into pMAL-p5x (New England BioLabs) by digestion and ligation steps. The positive plasmid was introduced into BL21 bacteria for protein expression. Crude bacterial lysates were filtered and loaded on the MBPTrap HP column (GE Healthcare). The fusion proteins were eluted using 10 mM maltose in equilibration buffer. Finally, MBP-hCR2 SCR1-2 proteins were purified further using size exclusion chromatography described earlier after dialyzed against 1× PBS and aliquoted to store at −80°C.

### SDS-PAGE and Immunoblot

Sodium dodecyl sulfate-polyacrylamide gel electrophoresis (SDS-PAGE) was performed on 10 or 4–12% gradient acrylamide gels. Generally, gels were ran for 1 h at 160 V. Total proteins were visualized by Coomassie brilliant blue staining for 1 h and destained until the background became transparent.

For immunoblotting, after SDS-PAGE, proteins were transferred onto polyvinylidene fluoride membrane (Millipore) for 2 h 30 min at 300 mA. Membranes were blocked in 5% non-fat milk (dissolved in TBST buffer containing 25 mM Tris, 250 mM NaCl, 0.1% Tween-20, pH 7.4) for at least 2 h at room temperature. For detection of soluble gp350-ECD_123_-6His, primary mouse anti-His antibody (Abmart) was diluted 1:2,000 and incubated with membrane at 4°C overnight following incubation with secondary goat anti-mouse antibody (1:20,000, Promega) conjugated to horseradish peroxidase (HRP) for 1 h at room temperature. For detection of Fc fusion proteins, the membranes were incubated directly with secondary goat anti-mouse antibody (1:20,000) for detection using ECL substrate (Advansta).

### Mouse Immunization

Five BALB/c female mice (6–8 weeks old) for each group were immunized with antigens intraperitoneally (i.p.) or intranasally (i.n.). For i.p. immunization, fusion proteins were adsorbed to Imject Alum adjuvant (Invitrogen) according to the manufacturer’s instructions, and two doses were tested, 1 or 20 µg. Primary immunization was performed after collection of preimmune sera at week 0 and followed by one boost at week 3. For i.n. immunization, 20 µg antigens (per mouse) was mixed with 25 µg CpG1826 (InvivoGen) and dissolved in 1× PBS, and the final volume was brought to 30 µl. Mice were immunized at weeks 0 and 2 after sedation induced by injection of 150 µl 5% chloral hydrate in PBS. Serum samples were collected after 0–5 weeks.

All mice were purchased from Beijing Vital River Laboratory Animal Technology Co., Ltd. (the joint venture of Charles River Laboratories in China). This study was carried out in accordance with the recommendations of the Institutional Animal Care and Use Committee at the Sun Yat-Sen University Cancer Center. The protocol was approved by the Institutional Animal Care and Use Committee at the Sun Yat-Sen University Cancer Center, and the animals were cared for in accordance with the institutional guidelines.

### Enzyme-Linked Immunosorbent Assay (ELISA)

For determination of serum titers, 96-well ELISA plates (Corning) were coated with soluble gp350-ECD_123_-6His (100 ng in 100 µl PBS per well) at 37°C for 2 h. After one wash, the plates were blocked using 1× ED buffer (0.5% casein sodium salt, 2% gelatin, 0.1% proclin-300, dissolved in 1× PBS) at 4°C overnight. Six 5-fold serial dilutions of serum samples were loaded on the plates (starting with dilution 1/100), and incubated for 1 h at 37°C, then washed with wash buffer. Secondary anti-mouse antibody conjugated with HRP (Promega) was diluted 1/20,000 and incubated for 45 min at 37°C. The signals were developed using EL-TMB kit (Sangon Biotech). Absorbance values at 450 nm were recorded, and the cutoff determined by OD values of pre-sera which was subtracted from experimental groups was set to 0.1. To determine the titers of gp350-specific Ig isotypes, the procedure was performed as before (46). Briefly, ELISA plates were coated with gp350-ECD_123_ as described earlier and incubated with diluted sera samples. Then, the Ig isotypes were detected using HRP-conjugated goat anti-mouse IgG1, IgG2a, IgG2b, IgG3, and IgA (1/10,000, Promega).

For competitive ELISA, mAb72A1 was directly conjugated with HRP using the HRP labeling kit (Proteintech). To quantify binding with mAb72A1-HRP, twofold serial dilution (starting at 1/100) were loaded on the plates coated with soluble gp350-ECD_123_-6His. The antibody dilution yielding an absorbance of 1.0 (i.e., 1/25,600) was chosen for competition tests. For competition, twofold serial dilutions of serum samples (starting at 1/50) were loaded on antigen-coated ELISA plates and incubated at 37°C for 1 h. Then, mAb72A1-HRP (dilution 1/25,600) was loaded on the plate and incubated at 37°C for 30 min, followed by signal development using the EL-TMB kit (Sangon Biotech). The competitive ability of serum samples against mAb72A1 was calculated using this equation: Percent of Inhibition % = [OD_(−serum samples/+72A1)_ − OD_(+serum samples/+72A1)_]/OD_(−serum samples/+72A1)_.

To characterize the binding of mAb72A1 to various gp350 fusion proteins, the fusion proteins were coated on the ELISA plates as described earlier. 24 twofold dilutions of mAb72A1-HRP were added and incubated with gp350 fusion proteins at 37°C for 45 min developed using EL-TMB kit.

To detect the binding with MBP-hCR2 SCR1-2, ELISA was performed as previously reported (45).

### Determination of Molecular Weight Using Analytical Ultracentrifugation (AUC) Analysis

Sedimentation velocity experiments were used to determine the *S* value and molecular weight of the purified recombinant protein samples at 20°C on a Beckman XL-A analytical ultracentrifuge equipped with absorbance optics and an An60-Ti rotor. The samples were diluted to 1 OD at 280 nm. PBS was used as the reference buffer. The rotor speed was set at 35,000 rpm for these samples. The sedimentation coefficient was obtained using the c(s) method with the Sedfit software, which was kindly provided by Dr. P. Schuck at the National Institutes of Health (http://www.analyticalultracentrifugation.com). And the molecular weight was calculated using the c(M) module of the Sedfit software.

### Surface Plasmon Resonance (SPR)

Surface plasmon resonance-based antibody competition was conducted on a Biacore T200 instrument (GE Healthcare) as described previously (32). Briefly, soluble gp350-ECD_123_-6His was immobilized on the CM5 sensor chip (GE Healthcare) by amine coupling at pH 5.5 until the response unit (RU) value reached ~500. Then, 50 µl of the serum samples (1/40 dilution) was injected at 30 µl/min, and the RU values were recorded as RU of serum. The chip was regenerated by one injection (50 µl) of 3 M MgCl_2_. Then, 50 µl (100 µg/ml) of mAb72A1 was injected at 30 µl/min before injecting of serum samples, and subsequently, the chip was regenerated by MgCl_2_. The RU values were documented as RU of mAb72A1. Competitive binding ability of serum samples to gp350 was calculated by an equation: Competitive percentage% = (RU of sera − RU of mAb72A1)/RU of sera.

For affinity kinetics analysis between recombinant gp350 and mAb72A1 or MBP-hCR2 SCR1-2, the three recombinant gp350s were immobilized on the CM5 sensor chip (GE Healthcare) at pH 5.5 with low RU value to avoid avidity effect. Then, mAb72A1 or MBP-hCR2 SCR1-2 was injected at 30 µl/min, and the chip was regenerated by 10 mM glycine–HCl, pH 1.5. Surface bound-kinetics fit (1:1 fit) was applied for data analysis, and χ^2^ less than 10% of *R*_max_ value was considered good.

### Infection Blocking Assay

To test the sera’s ability to block EBV infection of B-lymphocytes, an infection blocking assay was performed according to previous reports (24). Briefly, EBV_GFP_ was prepared using virus producing cell line, CNE2-EBV_GFP_ carrying Akata-EBV-GFP, and aliquoted 100 µl per tube. Immunized sera or mAb72A1 were mixed gently with 100 µl EBV in 1.5 ml Eppendorf tube and incubated for 1 h at room temperature. Then, ~1 × 10^5^ Akata negative cells (without latent EBV) were suspended with the mixture of antibodies and virus and incubated for 3 h at 37°C. Following the incubation, the cells were pelleted by centrifugation and washed once using 1 ml PBS. Then, cells were grown in 1 ml RPMI1640 plus 10% FBS in 12-well plates for 2 days. Uninfected cells incubated with RPMI1640 media were used as negative controls, and cells incubated with EBV, in the absence of immune serum, were included as positive controls.

After 2 days, the cells were collected and washed once with PBS. The EBV infection rate was determined using FACS analysis.

### Statistics

All statistical analyses were conducted with GraphPad Prism version 5. *p*-Values were generated by one-way ANOVA unless specified otherwise. *p*-Values of ≤0.05 were considered statistically significant.

## Results

### Construction and Production of Secreted Fc Fusion Proteins

The fusion with Fc domain of immunoglobulin (IgG) has been shown to dramatically enhance the immunogenicity of antigens due to dimerization and high affinity for FcRn (37, 47). To enhance the immunogenicity and protection potential of EBV gp350, we generated two new Fc-based constructs. The first construct contains the gp350-ECD_123_ domains with the mIgG2a-Fc domain at its C-terminus. These two domains are separated by a (Gly_4_Ser)_3_ linker to ensure correct protein folding and function (Figure 1A). Since the immunogenicity of the full-length ectodomain of gp350 in the context of a fusion protein is not well characterized, we designed an Fc-based construct with gp350-ECD_FL_. The gp64 signal peptide was introduced at the N-terminus for protein secretion, and a Kozak sequence (GCCACC) was included to enhance transcription. For purification of soluble gp350-ECD_123_, a 6× His tag was added at the C-terminus (Figure 1A).

For baculovirus production, Sf9 cells were transfected with recombinant bacmids for each construct. The fusion proteins were secreted into culture medium, and detected by Western blot and dot blot. For soluble gp350-ECD_123_, detection with an anti-His antibody yielded a single band of ~70 kDa under either reducing or non-reducing conditions by Western blot (Figure 1B). This is consistent with gp350-ECD_123_ being a monomer. For gp350-ECD_123_-Fc_mIgG2a_, Fc-detection yielded a ~110 kDa band under reducing condition and a ~220 kDa band under non-reducing condition. This confirms the dimeric form of gp350-ECD_123_-Fc_mIgG2a_. For gp350-ECD_FL_-Fc_mIgG2a_, the apparent molecular weight under reducing conditions was ~200 kDa, and increased to ~450 kDa without reducing reagent. Under non-reducing conditions, smaller products are visible for gp350-ECD_FL_, which may indicate Fc-containing fragments resulting from partial degradation of the fusion protein. In dot blot, the conformation-dependent neutralizing anti-gp350 monoclonal antibody mAb72A1 specifically recognized all constructs, indicated that the functional region folded correctly (Figure 1C). To purify these fusion proteins, affinity chromatography and gel filtration were performed (Figure 1D). We obtained homogenous preparations for each recombinant protein with molecular weights in agreement with the calculated values (data not shown). Fusion proteins containing gp350-ECD_123_ were well purified while gp350-ECD_FL_-Fc showed faint unrelated proteins, which were not detected by Western blot (not shown). Overall, the protein analysis shows that the gp350 domains dimerized through Fc domain and were correctly folded and processed through the secretory pathway.

### Binding of Fc Fusion Proteins With nAb, mAb72A1

To assess the proper activity of the recombinant gp350 fusion proteins, we tested their binding to the CR2/CD21 receptor and their recognition by the nAb mAb72A1. CR2/CD21 as acts as a receptor for gp350 to mediate entry of EBV into B-lymphocytes (25, 26). mAb72A1 is a specific nAb against gp350, which blocks EBV infection of B-lymphocytes through binding to the gp350 region recognizing CR2/CD21. We used ELISA and SPR to characterize the affinity and binding kinetics between gp350 and CR2/CD21 or mAb72A1. Purified MBP-hCR2 SCR1-2 was obtained from BL21 bacteria (see Materials and Methods), and mAb72A1 was purified from culture supernatants of HB168 hybridoma cells (24).

**Figure 2:**
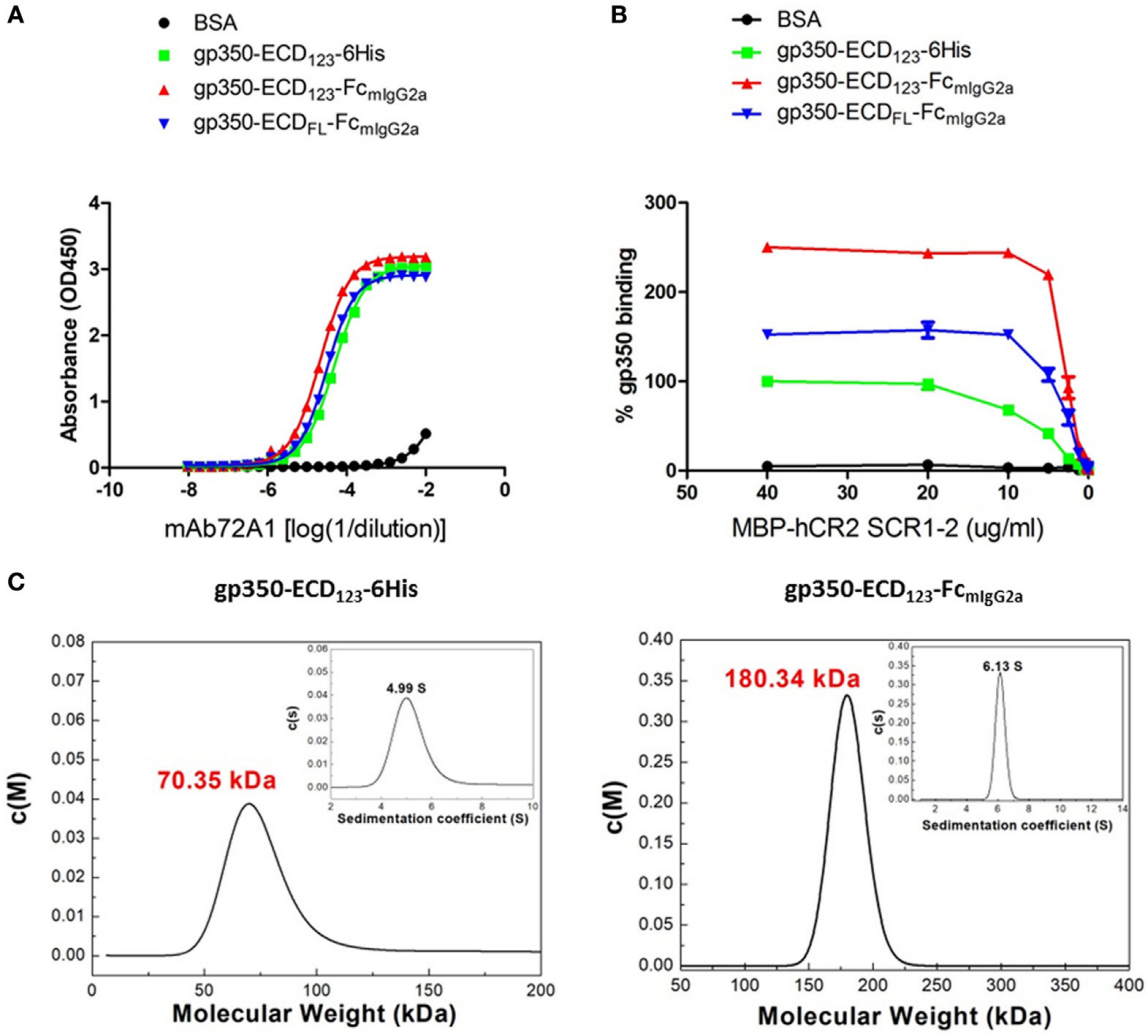
Molecular weight determination and binding kinetics analysis for recombinant gp350. **(A)** Binding of the indicated forms of recombinant gp350 to conformation-dependent neutralizing monoclonal antibody mAb72A1 by enzyme-linked immunosorbent assay (ELISA). BSA was included as negative control. **(B)** Binding of the indicated forms of recombinant gp350 to purified receptor MBP-hCR2 SCR1-2 by ELISA. Binding was normalized to maximum binding to monomeric gp350-ECD_123_-6His. BSA was included as negative control. The Fc fusion proteins bound CR2 more effectively than the monomeric gp350. **(C)** Analytical ultracentrifugation determination of molecular weight of the indicated forms of recombinant gp350 after purification. Sedimentation coefficients and molecular weights are indicated.

By ELISA, the neutralizing mAb 72A1 appears to detect all constructs equally well (Figure 2A). However, the refined binding kinetics analysis by SPR shows that the affinity for the dimeric gp350-ED_123_-Fc increased by about 100-fold compared with the monomeric gp350-ECD_123_ (Table 1). Both the gp350-ECD_FL_-Fc and gpECD_123_-Fc fusion proteins have the same affinity for the antibody (Table 1). Receptor binding was enhanced by fusing gp350-ECD_123_ to Fc. By ELISA, the dimeric constructs bound more soluble receptor than the monomeric gp350-ECD_123_ (Figure 2B), but this may reflect a stoichiometric effect rather than an affinity effect. SPR kinetic analysis shows that the affinity of the receptor for the dimeric gp350-ECD_123_-Fc increased by about threefold compared with the monomeric gp350-ECD_123_ (Table 1). The affinity of the full ectodomain Fc fusion protein for the receptors is somewhat similar to that of gp350-ECD_123_-Fc (Table 1). Overall, the *K*_D_ values of Fc fusion proteins were markedly smaller than that of the non-Fc fusion protein. This predicted higher affinity of Fc fusion proteins for the nAb mAb72A1 and for the receptor hCR2 likely results from the dimerization of Fc fragment.

**Table 1:** Affinity and binding kinetics of recombinant gp350 with mAb72A1 neutralizing antibody and hCR2 SCR1-2 receptor determined by surface plasmon resonance.

	*K*_D_ (M)	*k*_a_ (1/M s)	*k*_d_ (1/s)	χ^2^
**mAb72A1**				
gp350-ECD_123_-6His	1.525 × 10^−8^	5.254 × 10^4^	8.014 × 10^−4^	0.465
gp350-ECD_123_-Fc_mIgG2a_	1.324 × 10^−10^	6.904 × 10^5^	9.141 × 10^−5^	2.040
gp350-ECD_FL_-Fc_mIgG2a_	1.819 × 10^−10^	5.817 × 10^5^	1.058 × 10^−4^	2.490

**hCR2 SCR1-2**				
gp350-ECD_123_-6His	2.942 × 10^−7^	4.783 × 10^3^	1.407 × 10^−3^	0.560
gp350-ECD_123_-Fc_mIgG2a_	9.463 × 10^−8^	4.053 × 10^4^	3.835 × 10^−3^	0.610
gp350-ECD_FL_-Fc_mIgG2a_	6.847 × 10^−8^	1.755 × 10^4^	1.201 × 10^−3^	0.839

To confirm the dimeric conformation of gp350 fusion proteins caused by the Fc domain, the molecular weights of gp350 fusion proteins were determined using AUC analysis. As shown in Figure 2C, the molecular weight of gp350-ECD_123_-6His was 70.35 kDa, and that of gp350-ECD_123_-Fc_mIgG2a_ was 180.34 kDa, respectively. Thus, the Fc constructs have molecular weights corresponding to twice the size of the monomer and correspond to predicted values.

### Fc Fusion Antigen Were Notably More Immunogenic Than Monomeric gp350-ECD_123_-6His in the Presence of Alum or CpG Adjuvants

**Figure 3:**
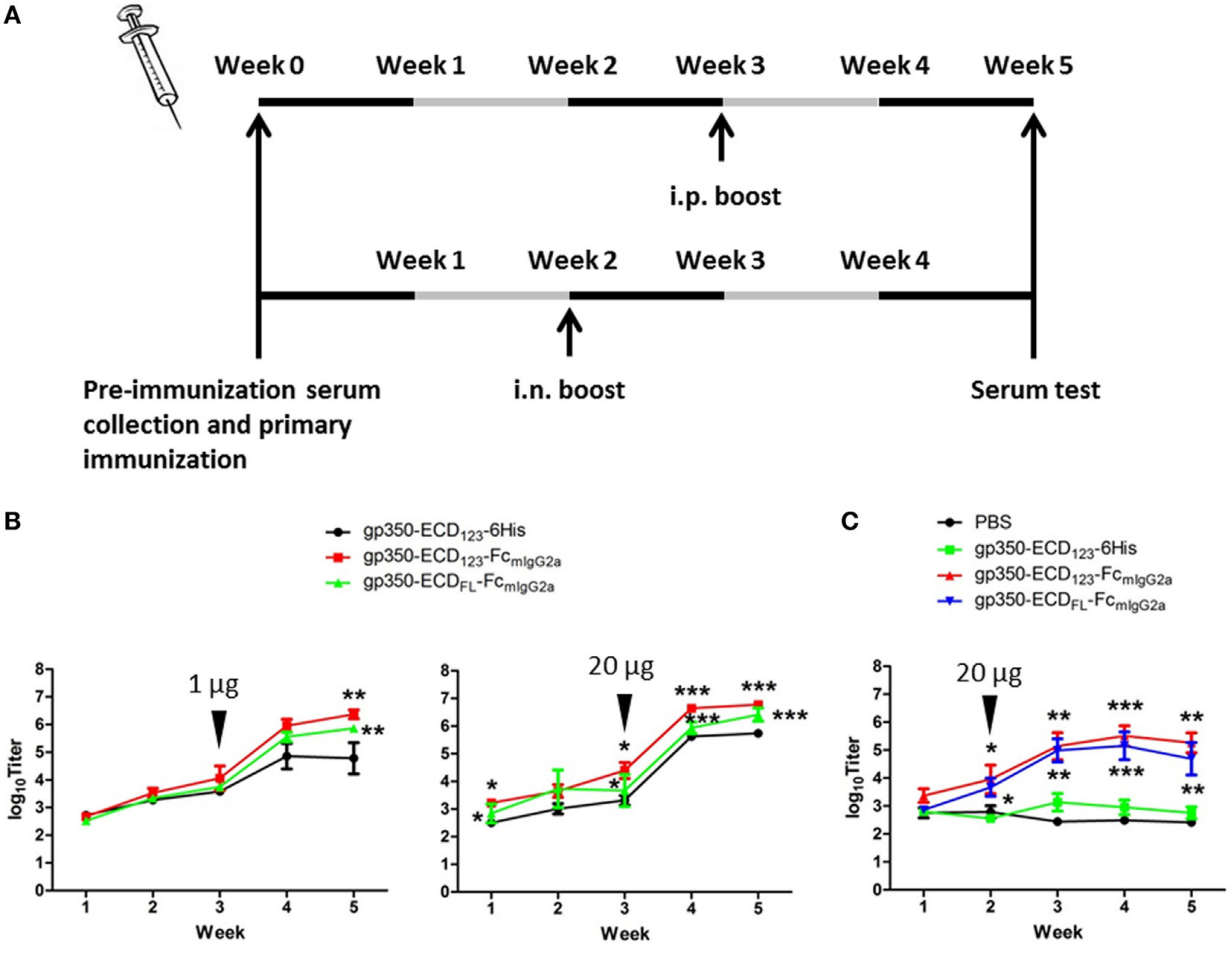
gp350 proteins fused with Fc_mIgG2a_ domain were notably more immunogenic than monomers in the presence of adjuvants. **(A)** Diagram of the immunization protocols. Mice were boosted on week 2 for i.n. group or week 3 for i.p. group. **(B)** Mice (five per group) were immunized i.p. with 1 µg (left panel) or 20 µg (right panel) recombinant proteins in Imject Alum adjuvant. **(C)** Mice (five per group) were immunized i.n. with PBS or recombinant gp350 proteins mixed with CpG1826. Black arrowheads indicate the times of boosts. Each data point represents mean ± SEM (*n* = 5). Significance (*p ≤ 0.05, **p < 0.01, and ***p < 0.001) between Fc fusion proteins versus monomer gp350 proteins is shown.

To determine the immunogenicity of Fc fusion proteins *in vivo*, BALB/c mice were immunized with gp350-Fc fusion proteins or monomeric gp350. Mice were injected and boosted i.p. with each antigen in the presence of Imject Alum adjuvant according to the schedule in Figure 3A. The specific anti-gp350 antibody titers were tested by ELISA against the monomeric gp350-ECD_123_. Serum titers from mice immunized with either gp350-Fc fusion protein were very significantly (*p* < 0.001) higher than that of the control group immunized with monomeric gp350 for both 1 and 20 µg doses (Figure 3B). The difference between monomeric and Fc-based gp350 antigen increased after a single boost for both doses tested. Considering that Fc fusion antigens could elicit an antibody response after mucosal immunization, we also immunized and boosted mice intranasally (i.n.) in the presence of CpG1826. Mice were boosted 2 weeks after the primary injection, and specific antibody titer against gp350 was determined 3 weeks later using ELISA (Figure 3A). As shown in Figure 3C, the serum titers of mice immunized i.n. with gp350-Fc fusion antigens were drastically higher than titers of mice immunized with monomeric gp350, which were similar to background signal likely due to non-specific serum IgG. After i.p. or i.n. injection, antibody titers in sera of mice immunized with gp350-ECD_FL_-Fc_mIgG2a_ appeared slightly, but not significantly, lower than titers in sera of mice immunized with gp350-ECD_123_-Fc_mIgG2a_. Overall, regardless of the immunization route, Fc-based gp350 antigens were more immunogenic than the native monomeric gp350.

### Immunization With Fc Fusion Antigens Potently Induced Production of nAbs

**Figure 4:**
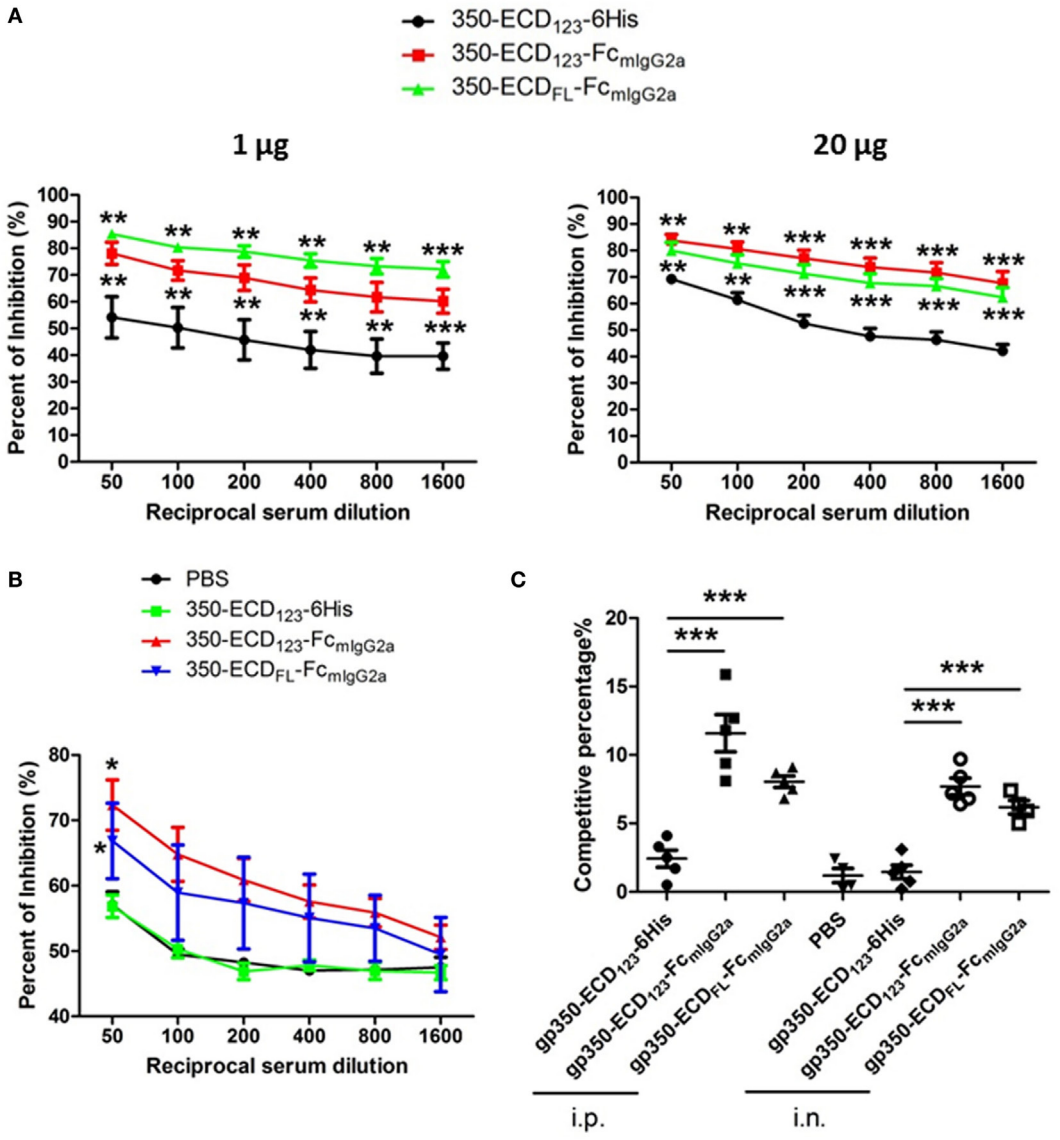
Immunization with Fc-based recombinant gp350 proteins elicits dramatically higher neutralizing titers than monomers. **(A)** Competition enzyme-linked immunosorbent assay (ELISA). BALB/c mice (five per group) were immunized i.p. with 1 µg (left panel) or 20 µg (right panel) of the indicated proteins in Alum adjuvant. Serum dilutions were used to block binding of neutralizing monoclonal antibody mAb72A1 to monomeric gp350 in competition ELISA. Percentage of inhibition of mAb72A1 binding is reported. **(B)** Competition ELISA. BALB/c mice (five per group) were immunized i.n. with 20 µg proteins assisted by CpG. Sera were analyzed for the ability to block mAb72A1 as in panel **(A)**. Each data point represents mean ± SEM (*n* = 5). **(C)** Competition by surface plasmon resonance (SPR). Neutralizing mAb72A1 was used to compete binding of serum immunoglobulin from mouse immunized with the indicated antigens to immobilized monomeric gp350 by SPR. The percentage of reduction of serum Ig binding is indicated. Each symbol represents the serum of one mouse (five mice per group), and the bars show mean ± SEM values. Significance (*p ≤ 0.05, **p < 0.01, and ***p < 0.001) between Fc fusion proteins versus monomer gp350 proteins is shown.

To address the presence of nAbs in sera of gp350 immunized mice, competition ELISA and SPR were carried out against the neutralizing mAb72A1. This competition approach demonstrates the presence of antibodies in serum, which share overlapping epitopes with known strong neutralizing monoclonal antibodies. This approach was validated for herpes simplex virus entry glycoproteins, where the ability of serum Ig to compete with specific nAbs directly correlated with the neutralizing ability of serum samples from infected and vaccinated individuals (48, 49). Wilson and Morgan showed a direct correlation between mAb72A1 competition and ability of mouse sera to neutralize infection (50). In the present competition ELISA, sera from mice immunized with gp350-Fc inhibited the binding of the neutralizing monoclonal antibody mAb72A1 to gp350 significantly better than sera from mice immunized with monomeric gp350 (Figures 4A,B). At low serum dilution, more than 80% of the binding of the nAb could be blocked by sera from mice, which received the gp350-Fc antigens. It did not escape us that sera from mice mock-immunized with PBS have high competition effect in this setting (Figure 4B). We decided to show the complete data rather than subtracting this background. Similar effect was seen previously in competition experiments (47). This indicates that i.n. immunization with monomeric gp350-ECD_123_ leads to low or no production of nAbs similar to mAb721A1.

The competition percentage was also evaluated by SPR for i.p. and i.n. immunizations (Figure 4C). Here, the competitive percentage represents the% reduction of serum Ig binding to gp350 following binding of the potent neutralizing mAb72A1. mAb72A1 reduced the gp350 binding ability of seric Ig from mice immunized i.p. with gp350-ECD_123_-Fc and gp350-ECD_FL_-FC by 12 and 8%, respectively. When i.n. immunization was performed, the competitive percentages were 8 and 6% for the respective dimeric antigens. By contrast, binding of serum Ig from mice immunized with the monomeric gp350-ECD_123_-6His was not affected by the neutralizing monoclonal antibody. These competition data show that the sera from mice immunized with gp350-Fc antigens, but not with monomeric gp350, contained antibodies that detect the neutralizing epitope specified by mAb72A1.

To determine whether the nAbs detected by competitive ELISA and SPR in Fc-350 sera have anti-EBV activity, the ability of these sera to block EBV infection of B-lymphocytes was tested. In this neutralization assay, EBV_GFP_ produced from CNE2 cells was incubated with diluted sera, and used to infect Akata cells (EBV negative). The neutralizing monoclonal mAb72A1 was included as a positive control.

**Figure 5:**
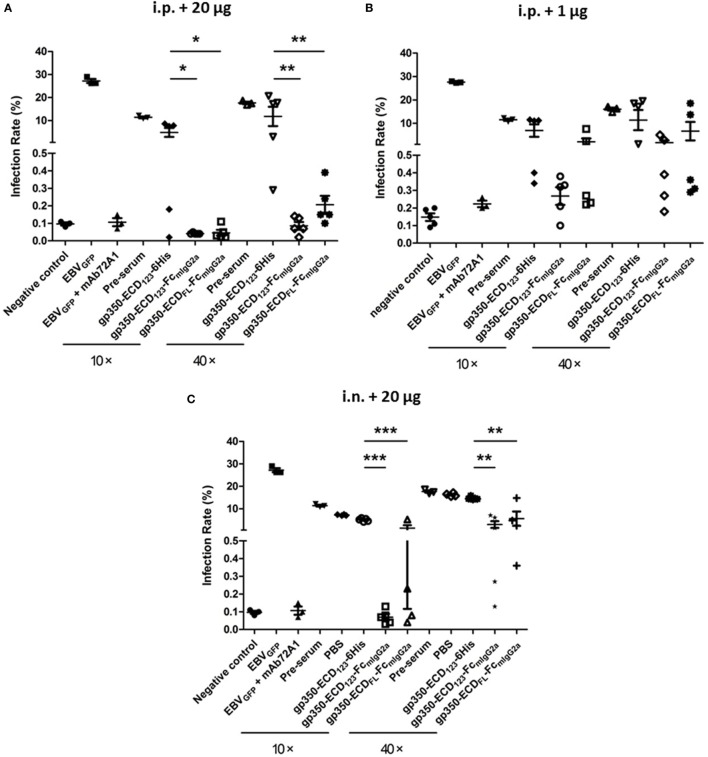
Neutralization of EBV_GFP_ infection by sera collected at week 5 post-immunization. Recombinant EBV_GFP_ was preincubated with 10× or 40× dilutions of sera from mice immunized with the indicated antigens. Virus was added to Akata cells, and GFP fluorescence was recorded as a measure of infection. The reported infection rates consist in the percentages of GFP positive cells as analyzed by flow cytometry. Virus infection was blocked by diluted sera developed through immunization. **(A)** Mice immunized i.p. with 1 µg antigen. **(B)** Mice immunized i.p. with 20 µg antigen. **(C)** Mice immunized i.n. with 20 µg antigen. Significance (*p ≤ 0.05, **p < 0.01, and ***p < 0.001) between Fc fusion proteins versus monomer gp350 proteins is shown.

Figure 5A shows the results for sera from mice immunized i.p. with 20 μg of the indicated antigens when diluted 10× or 40×. As expected, preimmune serum added only a limited effect compared with the negative control. Globally, immunization with the monomeric gp350-ECD_123_-6His did not elicit significant protection, although a couple of sera had more noticeable neutralizing effect compared with preimmune serum. By contrast, sera from mice immunized with either gp350-Fc construct, blocked infection efficiently at both dilutions (10× and 40×). The Fc-based constructs were significantly more potent than monomeric gp350 at both dilutions. The results of EBV neutralization assay with sera of mice that received a lower dose of antigen (1 μg) follow a similar trend (Figure 5B). However, the absolute neutralization potential was diminished, especially for the gp350-ECD_FL_-Fc, which was not significantly better than monomeric gp350. Sera from mice immunized with gp350-ECD_123_-Fc were only significantly protective when diluted 10-fold. At 40× dilution, it was limited to some but not all mice in each immunized group.

After intranasal immunization with 20 μg of antigen, gp350-ECD_123_-Fc, provided 100% neutralization with 10× diluted serum (Figure 5C). It was more efficient than gp350-ECD_FL_-Fc, but both Fc constructs were significantly better than monomeric gp350-ECD_123_-6His at both dilutions of sera.

We also tested whether there was a direct correlation between total anti-gp350 titers and ability of sera from different groups to block EBV infection of B cells. We found a strong linear correlation between total anti-gp350 titers (5 weeks post-immunization) and neutralizing activity (Figure S1 and Table S1 in Supplementary Material). The same correlation was observed at different dilution of sera (10×, 40×) used in these assays. The linear correlation occurred across all three forms of antigens for each route of immunization. Thus, Fc-based dimerization increased total titers and neutralizing titers in a similar manner. Overall, these data indicate that the immunogenicity of gp350-ECD_123_ was notably enhanced by fusion with an Fc domain.

### Immunization With gp350-ECD_123_-Fc_mIgG2a_ Elicited Markedly Higher Titers of IgG Isotypes and IgA

**Figure 6:**
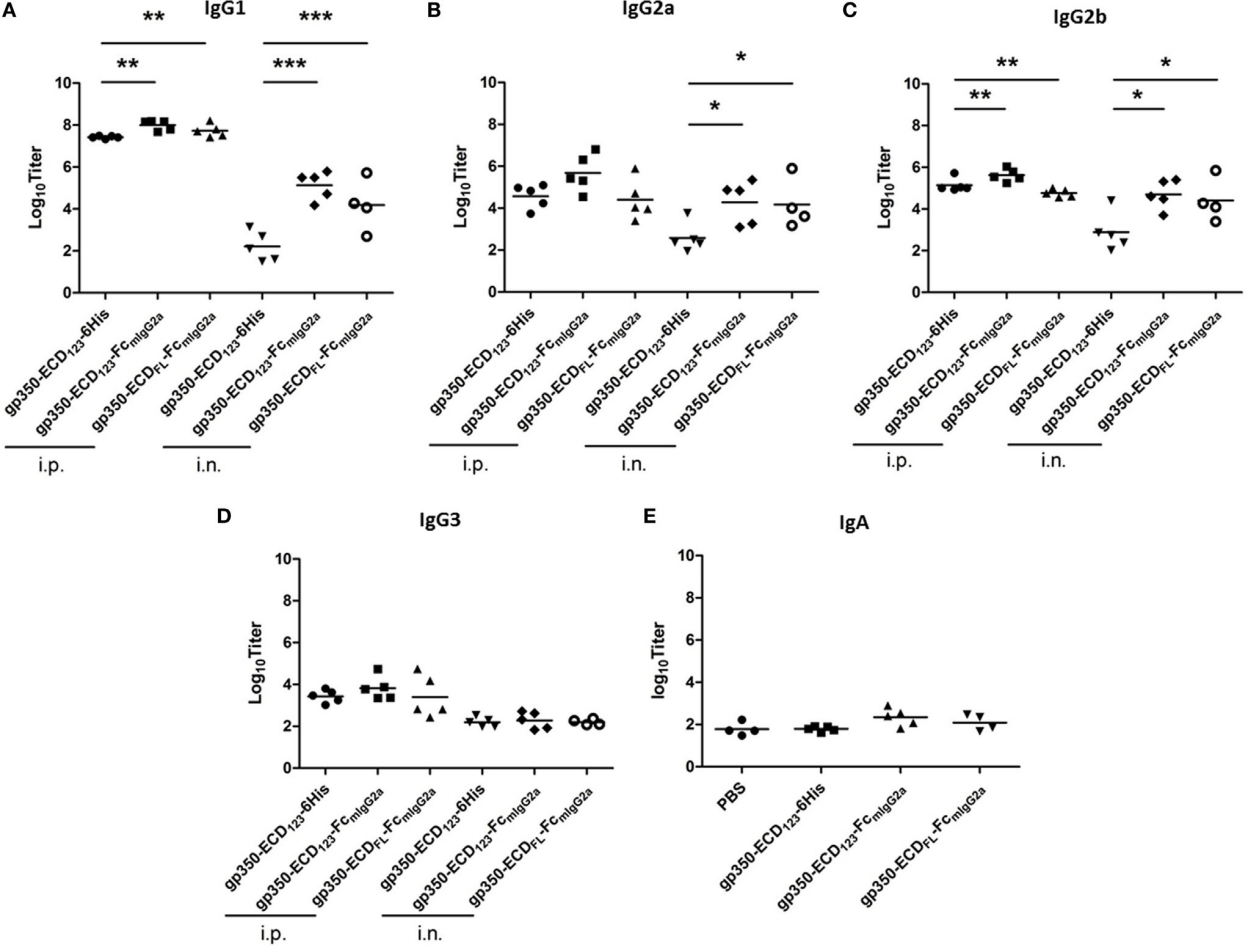
Specific titers of anti-gp350 IgG isotypes and IgA in sera are markedly elevated post-immunization with Fc-based gp350 proteins. **(A–D)** Titers of gp350-specific IgG subtypes were determined in sera (5 weeks post-immunization) of mice immunized i.p. or i.n. with 20 µg of the indicated antigen. **(E)** Anti-gp350 IgA serum titers from mice immunized i.n. with 20 µg of the indicated antigens. Each symbol represents an individual serum. Horizontal bars represent mean values. Significance (*p ≤ 0.05, **p < 0.01, and ***p < 0.001) between Fc fusion proteins versus monomer gp350 proteins is shown.

We compared the titers of different IgG isotypes between mice immunized with the various gp350 antigens (Figure 6). This analysis confirms that gp350-Fc is more immunogenic than monomeric gp350. The Fc-based antigens elicited more IgG1 and IgG2b than the monomeric counterpart gp350-ECD_123_-6His after i.p. immunization (Figures 6A,C). And after i.n. immunization, the gp350-Fc antigens induced markedly more IgG1, IgG2a, and IgG2b. Interestingly, all gp350 antigens elicited the same titer of IgG3, mostly after i.p. immunization (Figure 6D). The IgA serum titer was tested for mice immunized intranasally (Figure 6E). The specific IgA titers elicited by the immunization with Fc fusion proteins increased to some extent and follow the same trend as the IgG subtypes, although there is no statistical variation in this case. Enhancing the mucosal immunogenicity of gp350-Fc antigens may be improved by using adjuvants that are better adapted than CpG to elicit mucosal immunity [e.g., Poly(I:C), PEI]. Overall, these data confirm the highest immunogenicity of the Fc-based construct gp350-ECD_123_-Fc.

## Discussion

In our systematic study of the immunogenicity of the EBV envelope gp350 antigen, the full-length ectodomain (gp350-ECD_FL_) or functional domains 1-2-3 of the glycoprotein (gp350-ECD_123_) were fused to the Fc domain of mouse IgG2a. We showed that the Fc portion led to the dimerization of the normally monomeric gp350. Fusion with the Fc domain did not affect the native conformations of the receptor-binding domain and putative neutralizing epitopes. Indeed, the recombinant antigens with or without Fc domains have similar binding affinity and kinetics for the potent nAb, mAb72A1, and for the receptor, hCR2/CD21. More importantly, immunization with Fc-based gp350 fusion antigens elicited markedly more potent protection against EBV infection of B cells compared with monomeric gp350. This enhanced protection correlates with higher titers of nAbs in sera of mice immunized with the Fc-based gp350 constructs. When comparing both Fc-based constructs, it is not clear why gp350-ECD_123_-Fc was a more potent immunogen compared with the full ectodomain gp350-ECD_FL_-Fc. It may be because it contains fewer non-neutralizing epitopes compared with the full-length ectodomain. Alternatively, it may be caused by eliciting different Ig isotypes, although the contribution of each isotype to neutralization has not been determined. Finally, gp350-ECD_123_-Fc may be a more stable antigen with less degradation than gp350-ECD_FL_-Fc. In all our assays, we also observed a direct correlation between total anti-gp350 titers and inhibition of infection of B-cells (Figure S1 and Table S1 in Supplementary Material). This finding across three different forms of the gp350 antigens supports previous observations that anti-gp350 titers correlate better with inhibition of B-cell infection than anti-gp42 titers in humans (51).

The immunogenicity of monomer antigens is frequently enhanced through multimerization (52–55). In recent years, research on the development of EBV prophylactic vaccines has focused on the use of multimerization elements to improve immunogenicity. Cui et al. reported that a tetrameric gp350 linked by a leucine zipper domain and a trimer of gH/gL linked by the T4 fibritin foldon elicited more potent antigen-specific IgG and cytokine responses (56, 57). Kanekiyo et al. tested the immunogenicity of ferritin-based gp350 vaccine candidates in mice and non-human primates (32). Their results suggested that this approach induced potent nAbs. In the absence of direct comparison, one can only speculate about the most effective way of presenting gp350 as a dimer or a multimer. The Fc fusion approach has the advantage of having been validated in other human clinical trials (37, 43). Also, gH/gL-EBNA1 and gB-LMP2 fusion proteins have been showed to induce high antibody titers and EBV-specific T-cell responses in mice (58). Moreover, when fused with KLH, peptides, which have high affinity to EBV host cells, led to the production of neutralizing monoclonal antibodies against EBV (59, 60). Multimerization elements have shown many application prospects in these studies. However, the clinical safety and durability of these different approaches need further investigation, if possible in direct comparison, to be considered for clinical use.

The safety of Fc fusion protein as drugs has been widely validated in numerous clinical trials (37, 43). The Fc domain endows the recombinant antigens with a slower renal clearance rate due to dimerization and increased molecular weight. Through interaction with its corresponding receptor, FcRn, the plasma half-life of Fc fusion antigens was remarkably increased (37, 40, 42). Furthermore, the dimerization through Fc may induce a more efficient B cell receptor cross-linking (57). Based on the advantages brought by fusion with the Ig Fc domain, the immunity of antigens was potently enhanced (61). Besides, the antigens attached to Fc domain could be presented to APCs to elicit mucosal immunity. Altogether, the Fc-based version of an antigen is considered superior to its wild-type monomeric counterpart in preventing diseases acquired through mucosae (47, 62). EBV, which was described as one “kiss” virus, initiates infection of the host by infecting mucous epithelial cells (63). Consequently, this Fc-based approach serves as a new template to develop EBV prophylactic vaccine that target the initial infection of mucosa. Our data support the benefit of fusion of gp350 with the IgG Fc domain which has been preferred in vaccine design (i.e., mouse IgG2a or human IgG1). However, Fc domains from other Ig subtypes such as IgE and IgM, which have various affinities for different receptors, could be envisaged in further studies (37). For tests in non-human primates and for clinical use, human IgG1 Fc is preferred. The ability of hIGg1 to enhance immunogenicity of EBV gp350 is expected to be similar to that of the constructs based on mIgG2a presented here. Characterization of human Fc-based gp350 antigens is ongoing.

Previous gp350-based multimeric antigens have been tested in mouse models, including tetrameric gp350, virus-like particles, and ferritin nanoparticles (32, 57, 64). Due to different readout assays and protocols of injections, and because doses cannot be directly compared, any quantitative comparisons of total and neutralizing titers have to be interpreted very carefully. All these studies compare multimeric gp350 to a monomeric antigen, as we did here. This provides a framework to normalize and compare the efficacy of each approach. We found that Fc-based dimerization increased titers by 10- to 100-fold over monomeric gp350 (Figures 3B,C). This is in the range of titer increase reported for other multimeric forms of gp350 (32, 57, 64). Addition of Fc domains increased neutralization efficacy by about 100-fold (Figure 5). By comparison, the Fc-based approaches (20 µg i.n. or i.p.) appear as efficient ways of increasing anti-gp350 neutralization titers. Clearly, a side-by-side comparison with matched doses and analyses is needed to directly identify the most promising vaccine candidates in terms of efficacy and safety.

In previous clinical attempts, prophylactic vaccines targeting gp350 failed to block EBV infection in humans despite their ability to induce neutralizing Abs in rodent or non-human primates (17). In our study, when BALB/c mice were immunized with similar soluble gp350 monomers, only a few sera could block EBV infection of B-lymphocytes *in vitro*. By comparison, immunization with Fc-based recombinant gp350 increased neutralization titers and protection ability *in vitro*. This suggests that Fc-based gp350 antigens could provide a more potent protection against EBV infection, which may be due to the interaction of the Fc domain with its corresponding receptors. Furthermore, considering the complexity of the EBV virion envelope, a combination of gp350-Fc with other glycoproteins antigens could be included in candidate vaccine formulations. This may provide broader protection against EBV, which uses different subsets of glycoproteins to infect lymphocytes and epithelial cells. For instance, the potential application of gH/gL heterodimer and gB (a natural trimer) in EBV vaccine preparation has been discussed in previous reports since these essential glycoproteins are targeted by nAbs (56). However, very little information is available about the rational molecular design and optimal adjuvant formulation. Though some efforts have been made to investigate the usage of EBV gH/gL dimer and gB in vaccine research, it remains to be determined whether such antibodies would cut off the EBV infection in non-human primates.

In conclusion, our data with EBV gp350 suggest that the rational design to fuse the IgG Fc domain with the ectodomains of EBV glycoproteins could enhance immunogenicity, thereby providing more marked and universal protection against EBV infection. This strategy will guide further investigation to design EBV prophylactic vaccine, such as combinations of several EBV surface glycoproteins.

## Data Availability Statement

The authenticity of this article has been validated by uploading the key raw data onto the Research Data Deposit public platform (www.researchdata.org.cn), with the approval number RDDB2018000295.

## Ethics Statement

All experiments involving mice were approved by the Institutional Animal Care and Use Committee at the Sun Yat-Sen University Cancer Center, and the animals were cared for in accordance with the institutional guidelines. The mice were purchased from Beijing Vital River Laboratory Animal Technology Co., Ltd. (the joint venture of Charles River Laboratories in China).

## Author Contributions

YZ conceived the project and revised the manuscript; BZ designed and performed most of the experiments; XZ carried out collection of mouse serum samples and provided assistance for article supervision; CK contributed to data analysis and manuscript writing; SS carried out the AUC analysis experiment; HZ and LG cultured the insect cells; MX, LF, QF, and MZ provided assistance for some experiments; YX provided assistance on nuclei acid extraction. All the authors approved the final manuscript.

## Conflict of Interest Statement

The authors declare that the research was conducted in the absence of any commercial or financial relationships that could be construed as a potential conflict of interest.
