# Supplementary material for: Immunization With Fc-Based Recombinant Epstein–Barr Virus gp350 Elicits Potent Neutralizing Humoral Immune Response in a BALB/c Mice Model
Source: Front Immunol. 2018 May 1;9:932. doi: 10.3389/fimmu.2018.00932 (PMC5938345; doi:10.3389/fimmu.2018.00932)
Supplement: Supplementary file 1 [file Image_1.PDF]

i.p. + 20 μg

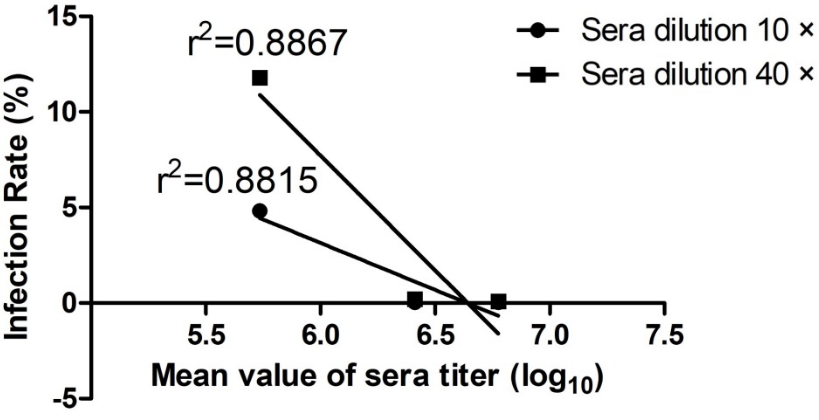

i.p. + 1 μg

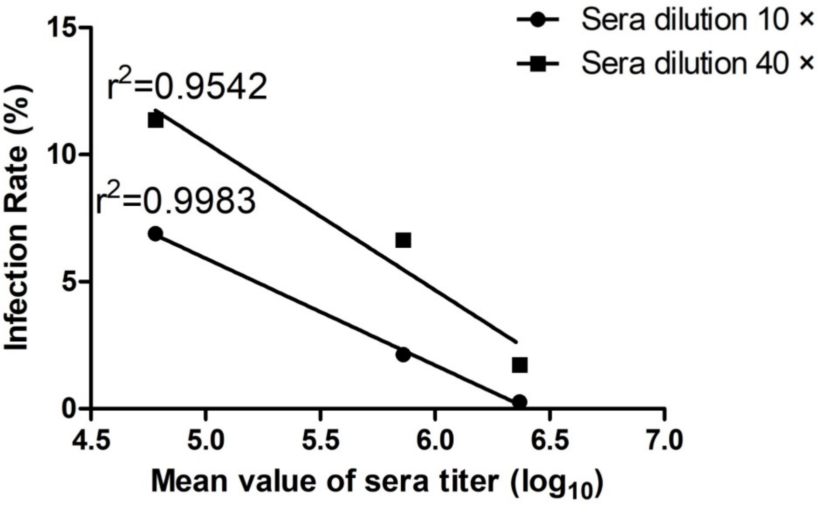

i.n. + 20 μg

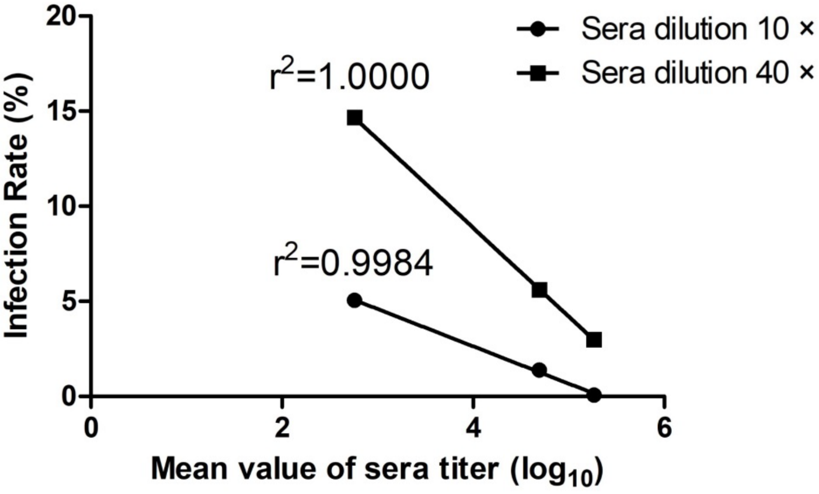

SUPPLEMENTARY FIGURE S1. Correlation analysis between specific sera titers against gp350 and corresponding neutralizing activity at Week 5. **(A)** Mice immunized i.p. with 20 μg antigen. **(B)** Mice immunized i.p. with 1 μg antigen. **(C)** Mice immunized i.n. with 20 μg antigen. The numeric data used for this analysis are summarized in Table S1. Correlation analysis by linear regression is performed using GraphPad Prism5, and $r^2 > 0.6$ is considered as good fitness.
