# Supplementary material for: Immunization With Fc-Based Recombinant Epstein–Barr Virus gp350 Elicits Potent Neutralizing Humoral Immune Response in a BALB/c Mice Model
Source: Front Immunol. 2018 May 1;9:932. doi: 10.3389/fimmu.2018.00932 (PMC5938345; doi:10.3389/fimmu.2018.00932)
Supplement: Supplementary file 2 [file Table_1.PDF]

SUPPLEMENTARY TABLE S1 Mean values of total specific sera titer against gp350 and corresponding neutralizing activity at Week 5.

**i.p. + 20 μg**			
	**Sera titer (log_{10})**	**Infection rate (%) 10 ×**	**Infection rate (%) 40 ×**
gp350-ECD_{123}-6His	5.735	4.828	11.798
gp350-ECD_{123}-Fc_{mIgG2a}	6.775	0.042	0.086
gp350-ECD_{FL}-Fc_{mIgG2a}	6.411	0.046	0.206
**i.p. + 1 μg**			
	**Sera titer (log_{10})**	**Infection rate (%) 10 ×**	**Infection rate (%) 40 ×**
gp350-ECD_{123}-6His	4.780	6.894	11.374
gp350-ECD_{123}-Fc_{mIgG2a}	6.369	0.268	1.736
gp350-ECD_{FL}-Fc_{mIgG2a}	5.861	2.136	6.64
**i.n. + 20 μg**			
	**Sera titer (log_{10})**	**Infection rate (%) 10 ×**	**Infection rate (%) 40 ×**
gp350-ECD_{123}-6His	2.756	5.052	14.676
gp350-ECD_{123}-Fc_{mIgG2a}	5.264	0.07	2.996
gp350-ECD_{FL}-Fc_{mIgG2a}	4.690	1.395	5.6025
